# Anesthetic management of a child with phosphomannomutase-2 congenital disorder of glycosylation (PMM2-CDG)

**DOI:** 10.1186/s40981-017-0080-y

**Published:** 2017-02-10

**Authors:** Wataru Sakai, Yusuke Yoshikawa, Yasuyuki Tokinaga, Michiaki Yamakage

**Affiliations:** Department of Anesthesiology, Sapporo Medical University School of Medicine, West16, South1, Chuouku, Sapporo, Hokkaido Japan

**Keywords:** Glycosylation, Neuromuscular blocking agents, Muscle hypotonia

## Abstract

**Background:**

Glycosylation is one of the major posttranslational modifications of proteins and it is essential for proteins to obtain normal biological functions. Congenital disorders of glycosylation (CDGs) are very rare genetic disorders that lack enzymes needed for glycosylation. Phosphomannomutase-2 (PMM2)-CDG is the most common type of CDG. CDGs can cause a wide variety of clinical symptoms in almost every organ system. Muscular hypotonia is often present in patients with CDGs and is one of the most notable problems for anesthetic management because the susceptibility to nondepolarizing neuromuscular blocking agents (NMBAs) in patients with CDGs is unknown.

**Case presentation:**

The patient was a 17-month-old boy who weighed 6.5 kg and was 71 cm tall. He presented for strabismus surgery. He had muscular hypotonia, mental retardation, hepatic dysfunction, mild cerebellar hypoplasia, and some dysmorphic features including inverted nipples and abnormal subcutaneous fat distribution of the hips. Gene analysis revealed a compound heterozygous mutation in the gene encoding PMM2 and the patient was diagnosed as having PMM2-CDG. General anesthesia was performed with sevoflurane, nitrous oxide, and rocuronium. Neuromuscular monitoring was performed during anesthesia using train-of-four (TOF)-Watch^®^ (MSD, Japan). As postoperative analgesia, the surgeon performed sub-Tenon’s anesthesia. We did not use any intravenous analgesic. After completion of the operation, residual rocuronium was competed by administration of sugammadex. The patient gained consciousness and spontaneous breathing was established shortly thereafter, and the trachea was smoothly extubated. He was transported to an inpatient ward and was discharged to his home the next day without any complication.

**Conclusions:**

We performed safe anesthetic management in a 17-month-old boy with PMM2-CDG using rocuronium under neuromuscular monitoring.

A patient with PMM2-CDG may show nearly normal susceptibility to nondepolarizing NMBAs.

## Background

Glycosylation is one of the major posttranslational modifications of proteins and it is essential for proteins to obtain normal biological functions. Congenital disorders of glycosylation (CDGs) are very rare genetic disorders caused by genetic deficits of some of the enzymes that are essential for glycosylation. Phosphomannomutase-2 (PMM2) converts mannose-6-phosphate into mannose-1-phosphate. PMM2-CDG, caused by PMM2 deficiency, is the most common type of CDG [1]. Although only about 700 patients with PMM2-CDG have been reported in the world [1], there may be many undiagnosed or misdiagnosed patients with CDG because of the lack of recognition of these disorders.

Glycosylation occurs in every living cell, and hence, CDG can cause abnormalities in nearly every organ system including mental retardation, hypertrophic cardiomyopathy, epilepsy, muscular hypotonia, hepatic dysfunction, coagulopathy, and endocrinopathy [1]. There is a high childhood mortality rate of approximately 25% in PMM2-CDG patients due to severe infection or organ failure [2]. We herein report anesthetic management of a PMM2-CDG pediatric patient with muscular hypotonia and hepatic dysfunction. Muscular hypotonia is one of the most notable problems for general anesthesia because neuromuscular blocking agents (NMBAs) can have an unexpectedly prolonged effect in patients with hypotonia. Since there have been very few reports about anesthetic management of patients with CDG [3, 4], the susceptibility of patients with CDG to nondepolarizing NMBAs remains unclear. This is the first case report about anesthetic management of a patient with PMM2-CDG using rocuronium under neuromuscular monitoring.

## Case presentation

The patient was a 17-month-old boy who weighed 6.5 kg and was 71 cm tall. He presented for strabismus surgery. He had muscular hypotonia, mental retardation, hepatic dysfunction, mild cerebellar hypoplasia, and some dysmorphic features including inverted nipples and abnormal subcutaneous fat distribution of the hips. He could move his extremities and head weakly, but he could not roll over and hold his head up. Although the patient had a sucking disorder due to muscular hypotonia, it was relatively mild and he did not need a tube for feeding. There was no preoperative history of aspiration pneumonia or sleep apnea at home. He was being administered ursodeoxycholic acid orally for treatment of his hepatic dysfunction.

Gene analysis revealed a compound heterozygous mutation in the gene encoding PMM2, and the patient was diagnosed as having PMM2-CDG. A preoperative laboratory examination revealed elevated transaminases (alanine aminotransferase and aspartate aminotransferase levels of 302 and 387 U/L, respectively).

After arrival in the operating room, an electrocardiogram and pulse oximeter were set up and general anesthesia was induced with a mixture of 5% sevoflurane, 50% nitrous oxide, and 50% oxygen. A 24-G intravenous line was inserted into a vein of the back of his hand. Neuromuscular monitoring was performed during anesthesia using train-of-four (TOF)-Watch^®^ (MSD, Japan) by stimulating the ulnar nerve of the forearm and sensing at the thumb. After calibration of TOF-Watch^®^, 2 mg (0.3 mg/kg) of rocuronium was first administered and TOF count 2 min later was 1/4. Then, 1 mg of rocuronium (total dose of 0.46 mg/kg) was added and TOF count 1 min later was 0/4. The trachea was intubated with a cuffed endotracheal tube. General anesthesia was maintained with a mixture of 1–3% of sevoflurane, 50% of nitrous oxide, and 50% of oxygen. After administration of rocuronium, TOF count was measured every 10 min during anesthesia. TOF counts 10 to 30 min later were all 0/4. Forty minutes later, TOF count had reached 2/4 and TOF ratios were 32, 80, and 89% at 50, 60, and 70 min later, respectively. The surgeon performed sub-Tenon’s anesthesia with 1% of lidocaine for postoperative analgesia. General circulatory and respiratory conditions were stable during surgery.

After completion of the operation, administration of sevoflurane and nitrous oxide was discontinued and the patient was ventilated with 100% of oxygen followed by administration of 7 mg (1 mg/kg) of sugammadex. The patient gained consciousness and spontaneous breathing was established shortly thereafter, and the trachea was smoothly extubated. He was transported to an inpatient ward and was discharged to his home the next day without any clinical symptoms of respiratory complications including aspiration pneumonia or upper airway obstruction and worsening hepatic function including jaundice. There was no perioperative bleeding complication.

### Discussion

We experienced a case of general anesthesia in a PMM2-CDG patient with hypotonia. In addition to the usual considerations for general anesthesia in pediatric patients, we paid careful attention to the use of NMBAs under neuromuscular monitoring and safely managed the patient with no complications. We also needed to take special care for selecting anesthetics because the patient had hepatic dysfunction.

Our case indicates that a patient with PMM2-CDG may show nearly normal susceptibility to nondepolarizing NMBAs. In general, NMBAs should be used very carefully in patients with muscular hypotonia. In patients with CDG, it is known that the expression of postsynaptic acetylcholine receptor with normal function is reduced [5]. In addition, both type 1 and type 2 muscle fibers are hypotrophic and the normal pattern of myofibrillar array is lost [6]. These functional and morphological changes in postsynapses and muscle fibers cause hypotonia in patients with CDG. We administered a requisite minimum dose of rocuronium using neuromuscular monitoring. According to a previous report [7], the mean recovery times from 0.45 mg/kg of rocuronium during isoflurane anesthesia in infants aged 5 to 12 months measured as TOF ratio >70% was 43.7 (range: 33.8–72.0) minutes. According to another report [8], the mean recovery times from 0.3 to 0.6 mg/kg of rocuronium during sevoflurane anesthesia in children aged 2 to 7 years measured as TOF ratio >80% were 24 (±8) (range 9–45) and 50 (±22) (range 24–122) minutes. The recovery time from 0.46 mg/kg of rocuronium in our patient, aged 17 months, measured as TOF ratio >80% was between 50 and 60 min and seems to be consistent with those reports. Although the level of muscular relaxation was not checked continuously, the approximate recovery time in this case provides important information because there is no previous report about recovery time.

Hepatic dysfunction is also a common feature in CDG patients [1]. A previous study revealed that swollen hepatocytes and fibrosis were present in liver specimens of patients with PMM2-CDG [9]. We considered the use of neuromuscular monitoring during anesthesia to be absolutely necessary for CDG patients because rocuronium is eliminated from plasma mainly by hepatic intake and biliary excretion [10, 11]. Since hepatic dysfunction could alter the metabolism of various other medications associated with anesthetic management, including intravenous anesthetics, opioids, and acetaminophen, we needed to pay special care to the selection of medications. Nitrous oxide and sevoflurane were administered for induction and maintenance of general anesthesia, and a local anesthetic agent was chosen for postoperative analgesia with avoidance of opioids in this case. Long-acting opioids should be avoided or very carefully used in patients with hepatic dysfunction because the effect could be abnormally prolonged. They may also cause serious respiratory complications in patients with muscular hypotonia because respiratory depression induced by opioids and respiratory muscular hypotonia may work synergistically in the postoperative period. In more invasive surgeries, remifentanil may be useful for intraoperative analgesia [3]. Acetaminophen was also avoided in this case because of the fear of further deterioration of hepatic function caused by hepatotoxicity by acetaminophen.

It is generally considered that some respiratory complications including aspiration pneumonia and upper airway obstruction can occur during the perioperative period [12, 13]. Although there was no preoperative history of frequent regurgitation, aspiration pneumonia, or sleep apnea at home, the patient had a mild sucking disorder and hypotonia. Therefore, we considered that he had a mild risk of perioperative respiratory complications. We performed careful anesthetic management with the minimum dose of a NMBA under neuromuscular monitoring and no usage of opiates, and we safely managed the patient without any complications.

## Conclusions

We describe a case in which we safely performed general anesthesia in a patient with PMM2-CDG using rocuronium with neuromuscular monitoring. Our case indicates that a patient with PMM2-CDG may show nearly normal susceptibility to nondepolarizing NMBAs. Further study is needed for a better understanding of and management of patients with CDG.

## Abbreviations

CDG: Congenital disorders of glycosylation
NMBAs: Neuromuscular blocking agents
PMM2: Phosphomannomutase-2
